# Supplementary material for: Association of Maternal Gestational Vitamin D Supplementation with Respiratory Health of Young Children
Source: Nutrients. 2023 May 19;15(10):2380. doi: 10.3390/nu15102380 (PMC10223031; doi:10.3390/nu15102380)
Supplement: Supplementary file 1 [file nutrients-15-02380-s001.zip › nutrients-2386285-Supplementary.pdf]

Supplementary data

Supplementary Table S1: Congenital abnormalities (CIM10 codes).

Congenital abnormalities (CIM10 codes)	
Neurologic	Q00 – Q07
Cardiovascular	Q20 - Q28
Respiratory	Q30 - Q34
Cleft lip and palate	Q35 - Q37
Digestive	Q39 - Q44
Renal and urinary	Q60 - Q64
Osteo-articular, muscular and cutaneous	Q76 - Q81
Chromosoal and genetic	Q90 - Q99

Supplementary Table S2: Characteristics of study population according to maternal gestational vitamin D3 supplementation.

Paramètres	Overall<br>(n = 125,756)	Maternal Vitamin D3 supplementation<br>(n = 54,696)	No maternal Vitamin D3 supplementation<br>(n = 71,060)
Maternal age (years)			
<20	1612 (1)	610 (1)	1002 (1)
20-29	53,920 (43)	23,357 (43)	30,563 (43)
30- 39	64,709 (51)	28,243 (52)	36,466 (51)
≥ 40	5515 (4)	2486 (4)	3029 (4)
Pregnancy follow-up			
Inadequate	9319 (7)	2696 (5)	6623 (9)
Intermediate	20,824 (17)	8754 (16)	12,070 (17)
Adequate	95,613 (76)	43,246 (79)	52,367 (74)
CHCI	30,524 (24)	14,075 (26)	16,449 (23)
NDI (Affluant)	28,828 (23)	11,636 (21)	17,192 (24)
Obstetrical pathology	19,766 (16)	9085 (17)	10,681 (15)
Caesarean section	15,858 (13)	6746 (12)	9112 (13)
Birth season			
Spring-Summer	50,999 (40)	21,886 (40)	29,113 (41)
GA (weeks)			
36	2258 (2)	829 (1)	1429 (2)
37	6293 (5)	2540 (5)	3753 (5)
38	18,469 (15)	7715 (14)	10754 (15)
39	37,689 (30)	16,353 (30)	21,336 (30)
≥ 40	61,047 (48)	27,259 (50)	33,788 (47)
Female sex	62,012 (49)	26,966 (49)	35046 (49)
Birth weight			
AGA	102,274 (82)	44,685 (82)	58,039 (82)
SGA	11,479 (9)	4859 (9)	6620 (9)
LGA	11,553 (9)	5152 (9)	6401 (9)
Neonatal pathology	15,217 (12)	7111 (13)	8106 (11)
Respiratory disease	46,142 (37)	19,938 (36)	26,204 (37)

Data are n (%).AGA: birth weight adapted for gestational age, SGA: small for gestational age, LGA: large for gestational age; CHCI: complementary healthcare insurance coverage; NDI: neighborhood deprived index. Relationships for all characteristics and status of maternal VitD supplementation were significant (p<0.0001) for all, except for cesarean section, female sex and birth weight for which p=0.001.
